# Aneurysm of the Abdominal Aorta

**Published:** 1903-12-26

**Authors:** 


### ANEURYSM OF THE ABDOMINAL AORTA.

In lecturing upon this subject at Guy's Hospital Dr. J. H. Bryant[1] mentioned that out of 18,678 necropsies which had been performed at Guy's Hospital between the years 1854 and 1900, 325 were cases of aneurysm of the aorta, of which 54 were of the abdominal portion. Of these 54 cases 49 were men—*i.e.*, over 90 per cent.; 77 per cent. of the patients were between 21 and 50 years of age, and in 63 per cent. the patients were under 40. These figures do not therefore support the statement that aneurysm of the abdominal aorta is a disease of middle life.

The two main factors in its causation are syphilis and violent exertion, and in many instances these two factors are associated. Of the 49 cases mentioned above there was a history of venereal disease in 20, and as some of the earlier reports were meagre it is possible that a history of venereal troubles might have been obtained in a larger proportion. Eight of the patients were old soldiers, all of whom had suffered from syphilis. Next to syphilis, acute rheumatism and chronic alcoholism appear to be the commonest associated diseases, but it is not clear if these conditions have any influence in the causation of aneurysm of the aorta.

The varieties of aneurysm are: saccular, diffuse, dissecting, fusiform, and arterio-venous. Of these the saccular and diffuse forms are by far the commonest, and the arterio venous the rarest, there being only two of the latter out of the 54 cases analysed. The diffuse form results from the rupture of a saccular aneurysm into the adjacent tissues. The leakage may be gradual but progressive, so as to produce a large tumour. The saccular and diffuse forms may arise from any part of the vessel, but most frequently commence in the neighbourhood of the cœliac axis—in 67 per cent. of Dr. Bryant's cases. Fusiform aneurysms involve the lower part of the vessel, dissecting aneurysms, the whole of the abdominal and often a part or the whole of the thoracic aorta, while the arterio-venous are found at the lowest part, at or just above the bifurcation. Erosion of the bodies of the vertebræ is one of the chief effects of the gradual increase in size of the sac. The amount of destruction may vary from a superficial erosion to an almost complete destruction of the bodies of the lowest dorsal and upper lumbar vertebræ, leading to exposure of and pressure on the spinal cord and its nerve roots. A curious feature of this erosion of bone is the invariability with which the intervertebral cartilaginous discs escape destruction—the reverse of what takes place in spinal caries. According to the direction in which the aneurysm extends pressure symptoms may develop.

If death occurs as a direct result of the aneurysm, rupture is almost always found as the cause, though it may occasionally happen that death is due to exhaustion, caused by constant and intense pain. Of the fifty-four examples analysed by Dr. Bryant, rupture recurred into the retro-peritoneal tissues in 19, the peritoneal cavity in 12, the pleural cavity in 8, the mediastinum in 2, the pericardial sac in 1, and the mesentery in 1. Rupture has been observed into the hollow viscera, *e.g.* stomach and bladder. In more than 50 per cent. of the cases ante mortem clot is found in the aneurysm, and may vary from a thin layer to a laminated mass completely filling the sac.

The most characteristic physical signs are—a tumour with expansile pulsation, occasionally a thrill, and more frequently a systolic bruit. In addition there are the signs produced by pressure on adjacent structures. Pain is the commonest and usually the first symptom. It is very variable in its site, character, duration and severity, but is usually referred to the abdomen or the back, and is almost always increased by movements of the body.

With regard to diagnosis, Dr. Bryant remarks that abdominal aneurysm is much oftener diagnosed when it is not present than overlooked when it is present. The condition which is most commonly mistaken for it is an excessively pulsating aorta. This is much more frequent in women than in men, while in the former abdominal aneurysm is extremely rare (only four cases in 47 years at Guy's). The most reliable sign is the presence of an abdominal tumour with expansile pulsation. The treatment is surgical, and consists of the introduction of wire into the sac. It is preferable, Dr. Bryant believes, to combine the introduction of the wire with electrolysis according to the method of Corradi. This is done by connecting the positive pole of a continuous current battery with the wire in the sac, and the negative pole with a métal plate applied to the patient's back, and allowing a current of from 10 to 20 milliampères to flow for about an hour. By these means it is stated the wire will become corroded and a firm clot formed in its vicinity.

[1] Clin. Jour., Nov. 18 and 25.

